# Porous N- and S-doped carbon–carbon composite electrodes by soft-templating for redox flow batteries

**DOI:** 10.3762/bjnano.10.113

**Published:** 2019-05-28

**Authors:** Maike Schnucklake, László Eifert, Jonathan Schneider, Roswitha Zeis, Christina Roth

**Affiliations:** 1 Institute of Chemistry and Biochemistry, Freie Universität Berlin, Takustraße 3, D-14195 Berlin, Germany; 2 Karlsruhe Institute of Technology, Helmholtz Institute Ulm, D-89081 Ulm, Germany; 3 Karlsruhe Institute of Technology, Institute of Physical Chemistry, D-76131 Karlsruhe, Germany

**Keywords:** N- and S-doped carbon, porous electrode, redox flow battery, soft-templating approach, vanadium

## Abstract

Highly porous carbon–carbon composite electrodes for the implementation in redox flow battery systems have been synthesized by a novel soft-templating approach. A PAN-based carbon felt was embedded into a solution containing a phenolic resin, a nitrogen source (pyrrole-2-carboxaldehyde) and a sulfur source (2-thiophenecarboxaldehyde), as well as a triblock copolymer (Pluronic^®^ F-127) acting as the structure-directing agent. By this strategy, highly porous carbon phase co-doped with nitrogen and sulfur was obtained inside the macroporous carbon felt. For the investigation of electrode structure and porosity X-ray photoelectron spectroscopy (XPS), scanning electron microscopy (SEM), and nitrogen sorption (BET) were used. The electrochemical performance of the carbon felts was evaluated by cyclic voltammetry (CV) and electrochemical impedance spectroscopy (EIS). The N- and S-doped carbon electrodes show promising activity for the positive side reaction and could be seen as a significant advance in the design of carbon felt electrodes for use in redox flow batteries.

## Introduction

In recent years, vanadium redox flow batteries (VRFBs) have attracted significant attention as a promising large-scale system for storing excess energy from renewable sources like wind or solar energy [1–3]. The energy is stored in the form of vanadium containing electrolytes, which consist of V^2+/3+^ at the negative and V^4+/5+^ at the positive side. These are flowed through carbon materials, which are usually porous felts or carbon paper electrodes [4]. Carbon electrodes exhibit good stability and electrochemical conductivity in the acidic and corrosive electrochemical environment of the battery system. Moreover, they are comparatively inexpensive [5]. One disadvantage is their poor electrochemical activity, which makes an activation step necessary [6].

A common way to achieve higher activities is the thermal treatment of the commercial felts (400 °C, up to 30 h) [7]. Also acid treatment [8], electrochemical activation [9], catalytic decoration with metal oxides [10–11], as well as methods to increase the surface area of the felts [12–13] have been reported as possible ways to obtain enhanced activity. Several works described heteroatom doping that should provide more active centres for the vanadium redox reactions, and hence lead to a higher electrochemical activity [14–17]. But still details of the mechanism are lacking and contradictory suggestions can be found in the literature, as to which functional group promotes the VO^2+^/VO_2_^+^ redox reaction the most [18].

The application of templates is a commonly used strategy to introduce porosity into carbon materials. Depending on the utilized template one can distinguish between a hard-templating and a soft-templating approach [19]. In both cases, the template acts as a structure-directing agent forming an inverse copy of the template morphology. Much higher surface areas can be achieved through this procedure, which offers many benefits in the later application, for instance increased adsorption due to more active sites leading to a better electrocatalytic activity. A disadvantage of the hard-templating approach is the requirement of harsh conditions that are needed to remove, e.g., SiO_2_ spheres used as templates [20]. In this respect, the soft-templating approach is a good alternative. The procedure is facile, with just two steps needed, and it is possible to scale-up the method to industrial standards [21]. In earlier studies we reported on a salt-templating method, where we embedded a PAN-based carbon felt into a eutectic mixture containing zinc chloride and sodium chloride mixed with an ionic liquid as the carbon source [22].

Herein, we present novel composite electrodes that were synthesized utilizing the soft-template method inspired by Martinez de Yuso et al. from 2017 [23]. With this method we are able to reduce the cost for the precursor materials significantly. We are able to use pyrrole-2-carboxyaldehyde, instead of ionic liquids, as nitrogen source. Also it is much more environmentally friendly, since zinc chloride is no longer needed and replaced by the structure-directing agent Pluronic^®^ F-127. Pluronic is a self-assembly block copolymer, which has already been widely used as template in the synthesis of porous carbons [24]. Strong interactions between phloroglucinol and the surfactant lead to the formation of hydrogen bonds to the polyethylene chains of the polymer. In this fashion the porogen initiates a porous structure. It will be removed during the subsequent carbonization step [25].

The new composite materials have great potential to serve as electrodes in the VRFB, since they combine the desired properties of the two components, namely good electron conductivity and high surface area. The carbon fibers as supporting material possess a high electron conductivity, while the amorphous carbon coating provides the catalytic functionality.

## Results and Discussion

**Figure 1:**
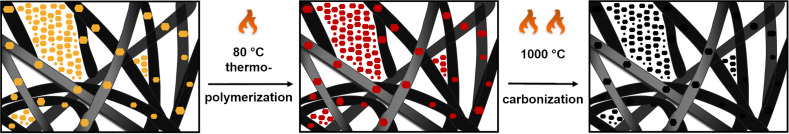
Schematic illustration of the synthesis route using the soft-templating approach to obtain porous carbon–carbon composite electrodes. The colours represent the changes of the appearance of the materials during the synthesis.

For the synthesis of nitrogen-doped carbon composite electrodes phloroglucinol was suggested as a carbon source whereas pyrrole-2-carboxaldehyde was utilized as a nitrogen source and the block copolymer Pluronic^®^ F-127 was used as porogen. In the co-doping process additional 2-thiophenecarboxaldehyde was employed as a sulfur source. The carbon–carbon composite materials were synthesized by soaking the felts in a solution containing the aforementioned precursors. After thermopolymerization under air a subsequent carbonization step under protective atmosphere, in which the porogen is removed, was performed to obtain highly porous carbon electrodes co-doped with nitrogen and sulfur. But not all of the formed carbon coating sticks to the surface of the felt fibers, some excess co-doped carbon material exists besides. This additional material is referred to as “bulk material” in the following text (Figure 1).

### Structural characterization

**Figure 2:**
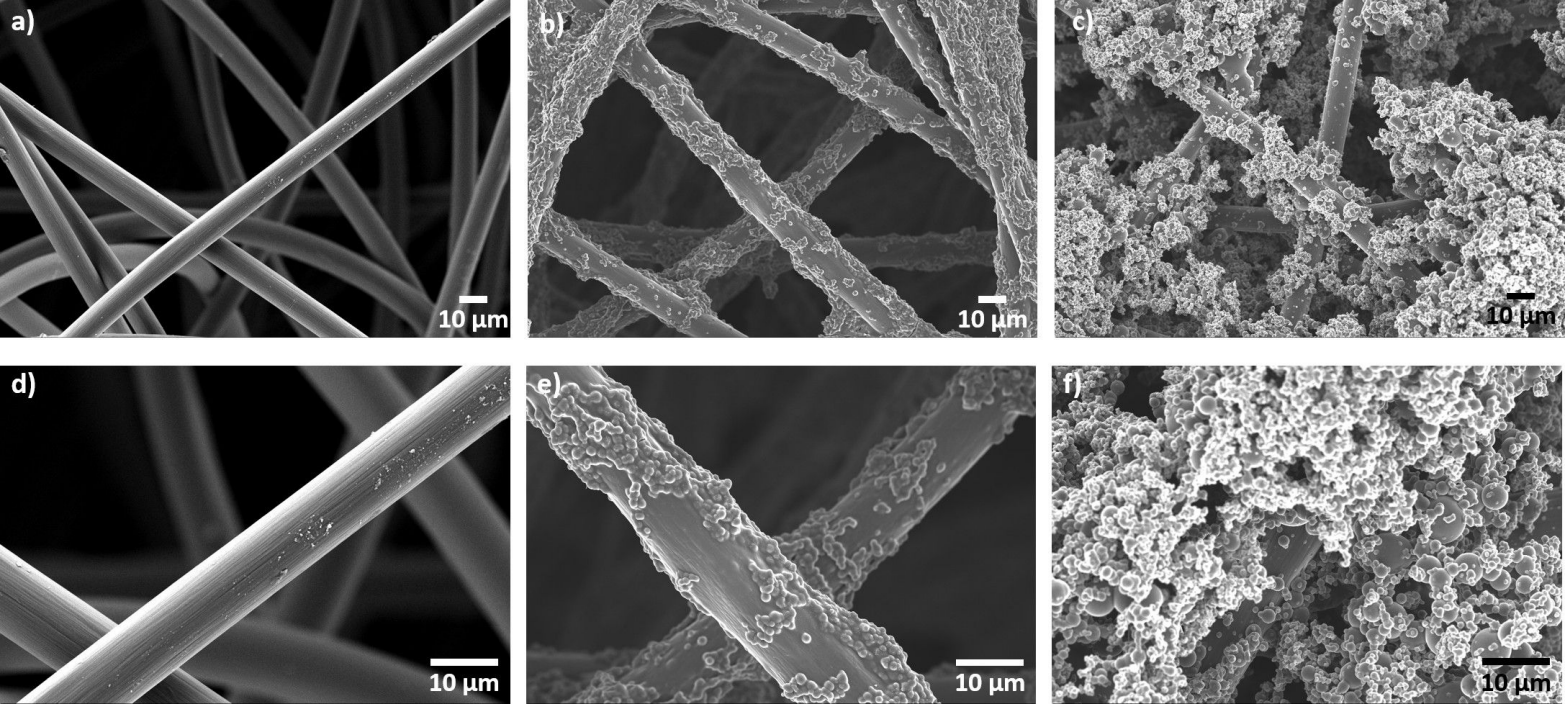
High-resolution images of carbonized carbon felt (left: a,d), N-doped carbon felt (middle: b,e) and S- and N-doped composite material (right: c,f) with 600× (upper row) and 1500× (bottom row) magnification.

For a detailed insight into the morphology of the electrode, SEM images of the carbonized sample, N-doped carbon felt and S- and N-doped composite material were taken at two different magnifications (Figure 2).

The fibers of the pristine felt appear arbitrarily oriented with a smooth surface. In comparison to that, the N-doped composite material as well as the composite electrode co-doped with nitrogen and sulfur are decorated with patches of agglomerated carbon material. Significant differences in the amount of deposited material can be observed. While the fibers of the co-doped composite electrode are covered completely and the space between fibers is filled up almost completely, the N-doped felt exhibits only partial coverage. It seems as if the carbon coating sticks more readily to the fibers after co-doping. But so far, we have not come up with a reasonable explanation for this observation.

**Figure 3:**
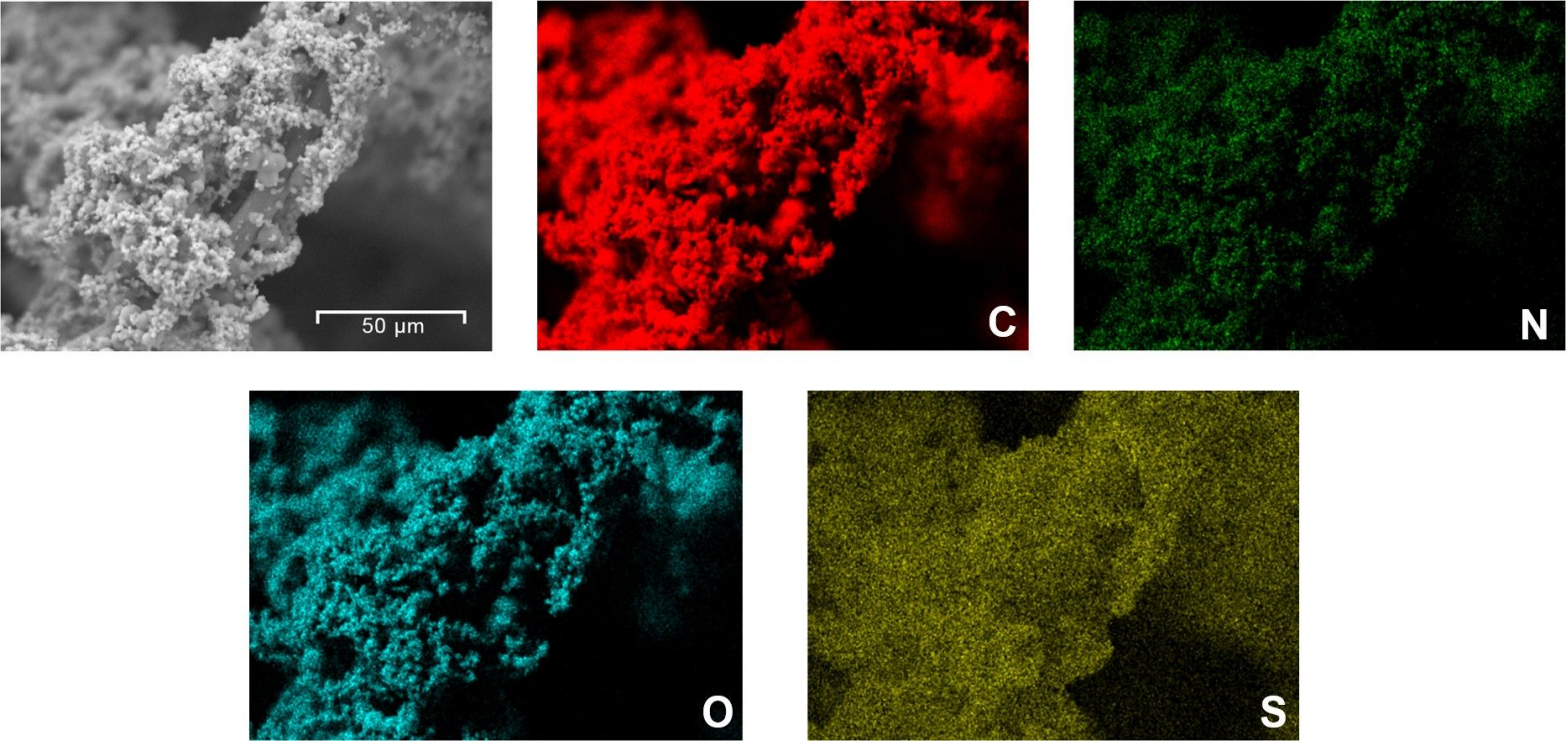
SEM image of the co-doped composite electrode and corresponding colored mappings of carbon, nitrogen, oxygen and sulfur.

Elemental mapping by scanning EDS was performed to investigate whether the co-doping was homogeneous and the results are shown in Figure 3.

The co-doped composite felt contains significant quantities of carbon, nitrogen, oxygen and sulfur. The EDX mappings verify the largely homogeneous distribution of all elements. Nitrogen doping as well as sulfur doping through the proposed soft-templating approach were successful.

**Figure 4:**
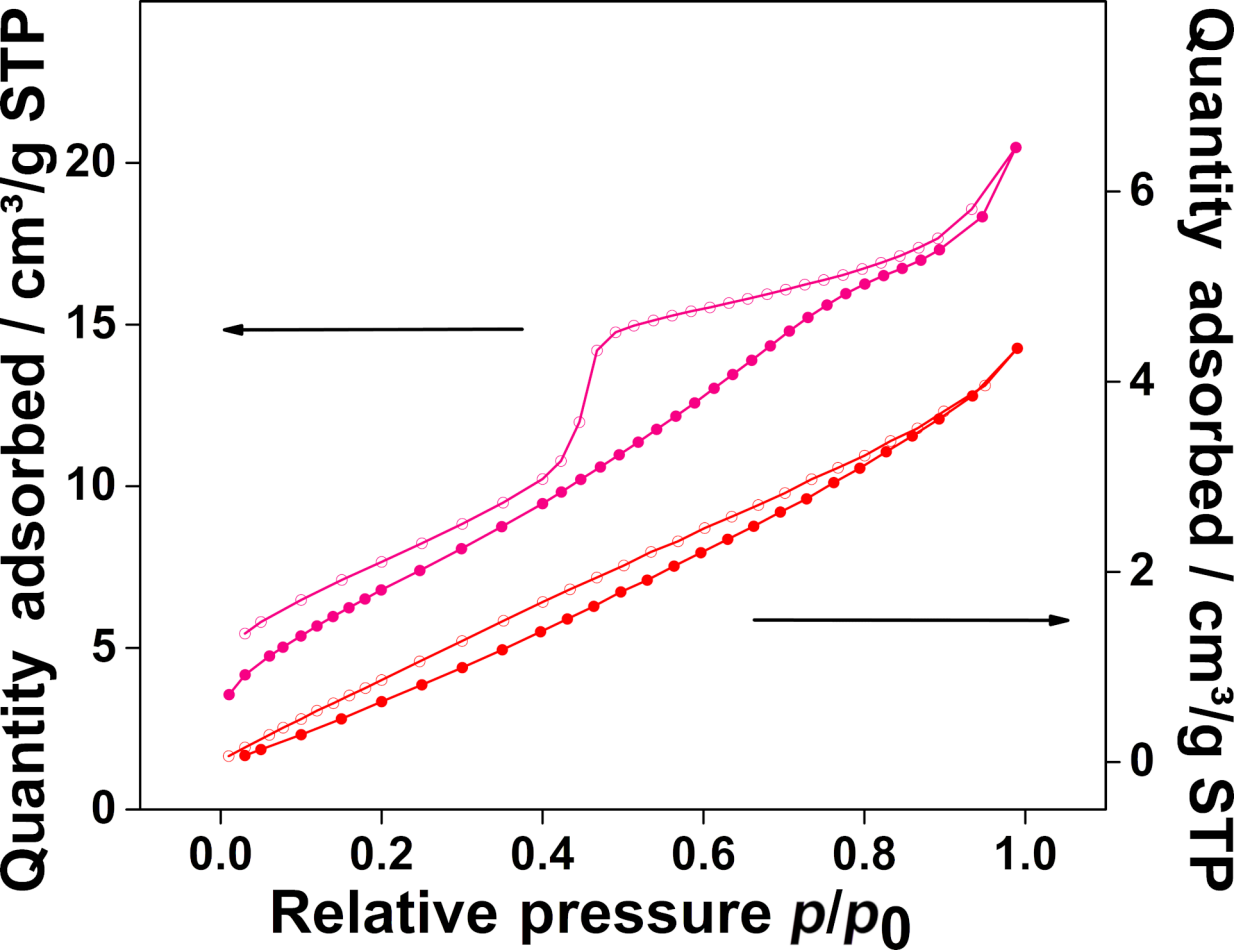
Nitrogen sorption isotherms of the synthesized composite electrode with nitrogen doping (upper line) and the carbonized carbon felt (lower line).

BET measurements were carried out to analyze the porosity of the carbon felts. In Figure 4, a comparison between the nitrogen sorption isotherms of the carbonized felt and the N-doped composite electrode is shown. The curves appear distinctively different with a significant hump observed for the doped electrode. With the soft-templating approach it is possible to enhance the surface area of the felts by a factor of up to 20 times in comparison to the undoped felt. The respective BET surface areas are listed in Table 1. It is found that the surface area increases as a result of the doping procedure for all felts heated up to 1000 °C. The enhanced specific surface areas of the synthesized composite electrodes (BET data) in combination with the SEM/EDX mappings indicate the successful functionalization of the pristine felt.

**Table 1:** BET surface areas of carbonized felt and composite electrodes heated up to 1000 °C.

	*S*_BET_/m^2^·g^−1^

carbonized (1000)	6.6
composite (1000 / N)	30.6
composite (1000 / N+S)	130.6

To define the elemental composition of the different carbon felt electrodes and the related bulk materials an elemental analysis was performed, with specific focus on nitrogen and sulfur content. Table 2 summarizes the elemental composition of the different felts. In accordance with our expectations the main component is carbon, followed by nitrogen and sulfur. The felts lose material during the carbonization step, and a trend can be observed that with increasing temperature the remaining nitrogen content as well as the sulfur content become reduced. The composite electrodes, however, contain more nitrogen than the bulk material. Note that the additional nitrogen originates from the PAN fibers.

**Table 2:** Elemental composition of the pristine and the carbonized felt as well as the composite electrode based on elemental analysis.

	C content/wt %	N content/wt %	S content/wt %	H content/wt %

pristine^a^	58.78	19.18	0.00	4.16
carbonized (800)	77.25	12.67	0.00	1.96
carbonized (1000)	91.77	5.92	0.00	0.04
composite (800 / N+S)	78.36	11.26	2.60	1.66
bulk material (800 / N+S)	84.85	6.37	4.33	1.46
composite (1000 / N+S)	85.59	4.26	2.23	1.18
bulk material (1000 / N+S)	88.95	2.90	2.34	1.28

^a^It is worth mentioning here that the pristine felt consists of only stabilized PAN fibers. The residual mass can be attributed to oxygen content or incomplete combustion of the samples.

**Figure 5:**
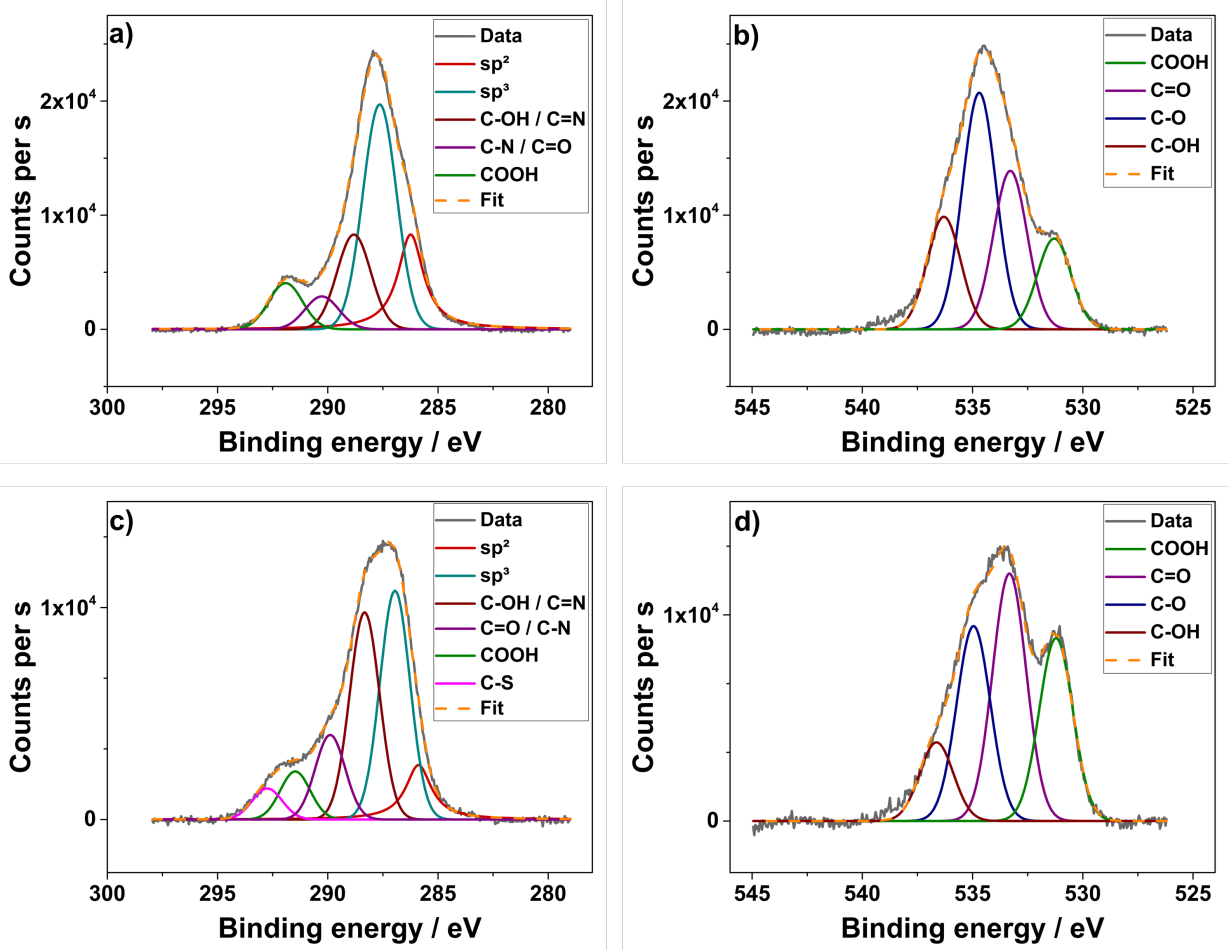
High-resolution XPS spectra including the fitting of the carbonized carbon felt (a,b) and the co-doped composite electrode (1000 / N+S; c,d): spectra of C 1s (left) and O 1s region (right).

XPS measurements were carried out to further examine the surface functional groups of the composite materials in contrast to the reference material (Figure 5). In agreement with recent literature the peaks were fitted to the most probable functional groups.

In the C 1s spectrum of the carbonized felt (Figure 5a) five individual peaks could be deconvoluted. There is one dominant peak, which could be assigned to sp^3^-hybridized carbon, while the other peaks at higher energy originate from different C–O bonding configurations. Also a small contribution could be allocated to sp^2^-hybridized carbon. The C 1s spectrum changes when nitrogen and sulfur atoms are doped into the carbon material (Figure 5c) [26–27]. For the composite electrode an additional peak associated with a C–S functional group could be observed. This additional peak indicates the presence of sulfur on the surface of the felt. Moreover, a change in the individual contributions could be noticed for the carbon felt co-doped with nitrogen and sulfur. It can be easily seen that the intensity of the sp^2^-hybridized carbon peak is reduced in favor of the peaks attributed to the functional groups.

The high-resolution spectrum of the O 1s region shows four peaks for both materials. The carbonized sample (Figure 5b) has one main contribution with a binding energy at 534.7 eV, corresponding to the C–O functional group. Two additional peaks at 533.3 eV and 536.3 eV could be assigned to C=O and C–OH, while the shoulder peak at 531.3 eV could be attributed to COOH. These signals could also be detected in the O 1s spectra of the composite electrode. However, the intensities of the different contributions change significantly. In contrast to the carbonized sample the co-doped material shows a significant increase in C=O and COOH content, whereas the amount of C–OH is slightly reduced. In the literature, it is frequently found that an increase in the functional groups on the surface of the fibers increases their hydrophilicity and hence their electrochemical performance [28–29]. We refrained, however, from quantitative analysis due to the inherent restrictions of fitting the rather broad C 1s and O 1s peaks with a multitude of individual contributions.

### Electrochemical characterization

**Figure 6:**
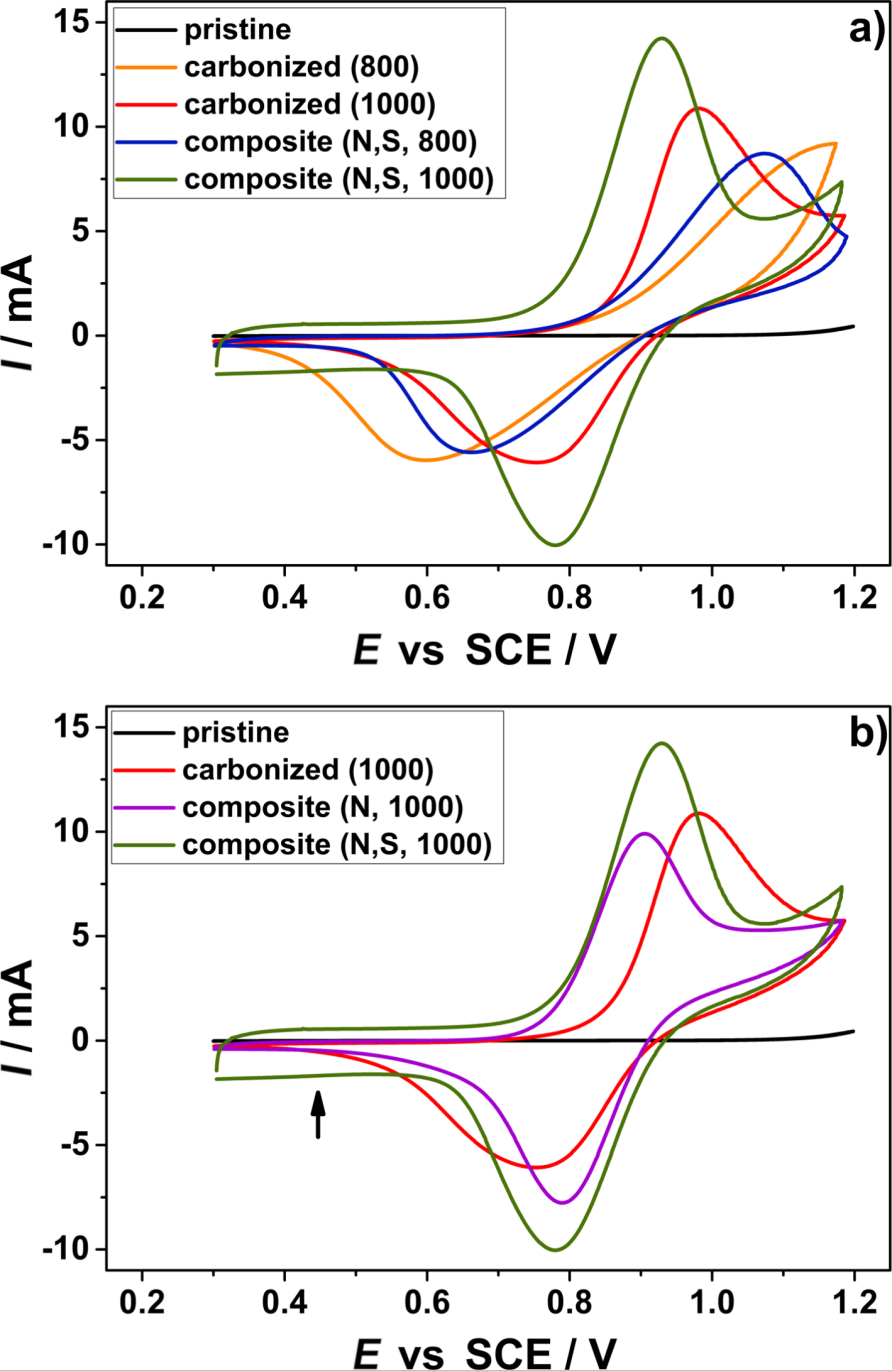
Comparison between the cyclic voltammetry curves of the N-doped (purple) and co-doped (blue, green) composite electrode as well as the carbonized (orange, red) and the pristine (black) felt for the positive half-cell reaction, (a) influence of the temperature used during the treatment and (b) influence of the doping method. The electrolyte consists of 2 M sulfuric acid and additional 0.2 mol/L vanadyl sulfate.

**Figure 7:**
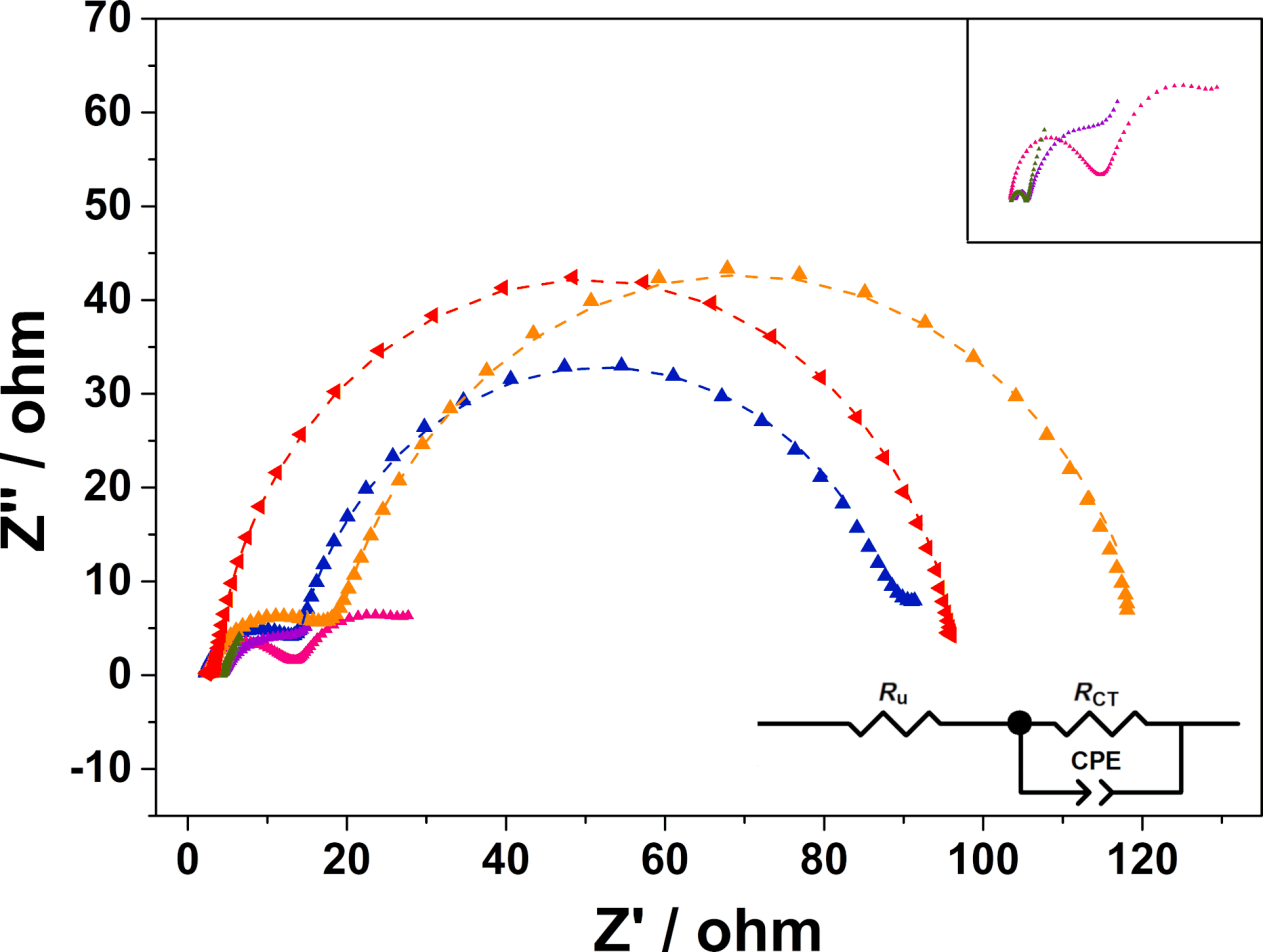
Nyquist plots showing the EIS data for the carbonized felts (orange, red) as well as for the composite electrodes heated up to 800 °C (pink, blue) and 1000 °C (purple, green) in the electrolyte solution. The data were fitted to the circuit model displayed in the graph and the simulated curves are shown as dashed lines. In the inset, the data range from 0 to 30 Ω is enlarged to show details of the N-doped composite electrode (pink, purple) and the co-doped sample (green).

The electrocatalytic activity of the composite electrodes for the positive redox reaction VO^2+^/VO_2_^+^ was investigated by CV (Table 3) and EIS. The CV curves of the respective carbon samples are shown in Figure 6 and the Nyquist plots are shown in Figure 7.

**Table 3:** Electrochemical data of the VO^2+^/VO_2_^+^ redox couple obtained from cyclic voltammograms.

	peak current/mA	peak position *E*_p_/mV	peak separation Δ*E*_p_/mV

carbonized (800)	—	—	—
	−6.0	597	
carbonized (1000)	10.9	982	228
	−6.1	754	
composite (800 / N)	8.2	1000	296
	−6.6	704	
composite (1000 / N)	9.9	906	116
	−7.8	790	
composite (800 / N+S)	8.7	1073	411
	−5.6	662	
composite (1000 / N+S)	14.2	929	149
	10.0	780	

The pristine felt does not show any catalytic activity for the positive side reaction at all, whereas the carbonized samples exhibit at least poor activity. For the felt carbonized at 800 °C no complete oxidation peak was measured. All samples follow the trend that with increasing temperature an increase in activity is observed (see Figure 6a). In comparison to samples heated up to only 800 °C, the felt carbonized at 1000 °C shows a significant shift of the peak positions resulting in a lower peak separation. In the literature it is often proposed that enhanced electrochemical activity could be ascribed to the presence of abundant heteroatom-containing functional groups [17,29]. Similar behavior could be noted here, with heteroatoms into the carbon felt an increase in activity is observed. Especially, the co-doped composite material possesses an excellent electrochemical performance. It provides high maximum currents combined with a small peak separation, and the maximum currents appear symmetrical. These parameters indicate a good reversibility, which is beneficial for a reliable and stable cycling of the battery. It is worth mentioning here that the increased double layer capacitance (DLC) in the region between 0.3 and 0.6 V vs SCE is further evidence of an enhanced surface area (marked with an arrow in Figure 6b). This is in good agreement with the BET measurements (Table 1). Due to the enhanced surface area, the amount of vanadium ions that can be adsorbed onto the surface increases, resulting in higher currents compared to the only carbonized samples. The obtained data suggest that the kinetics regarding the VO^2+^/VO_2_^+^ redox couple follow the order of carbonized < composite (N-doped) < composite (co-doped with N and S). This means that the redox reaction of the vanadium ions proceeds more readily on the composite electrodes than on the undoped reference materials.

Additional electrochemical impedance spectroscopy was performed to study the charge transfer of the VO^2+^/VO_2_^+^ redox reaction. The corresponding Nyquist plots are displayed in Figure 7. In the described circuit *R*_u_ stands for the solution resistance, which varies with the used electrode. The CPE is the constant-phase element which could be converted into the electric double-layer capacitance (*C*_DL_), whereas *R*_ct_ represents the charge transfer resistance. Compared to the carbonized samples the diameters of the semicircles of the composite materials are significantly reduced, directly reflecting the reduced charge transfer resistances of these electrodes (Table 4). Obviously, the addition of a porous carbon layer decreases the charge transfer resistance, so that the electrochemical reaction can proceed faster. In accordance with the CV measurements, the co-doped electrode possesses the largest double-layer capacity of the electrode/electrolyte interface, which is beneficial for the charge transfer of the positive side reaction [30].

**Table 4:** Values for the charge transfer resistance and the double-layer capacity received by fitting the impedance data with corresponding circuit model in Figure 7 and converting the CPE parameters following Hirschorn and co-workers [31].

	*R*_CT_/Ω	*C*_DL_/mF

carbonized (800)	104.0	0.383
carbonized (1000)	93.38	0.143
composite (800 / N+S)	78.19	0.376
composite (1000 / N+S)	53.89	310.0

The combined results of CV and EIS allow for the conclusion that an increased amount of functional groups on the surface of the electrode as well as an increased surface area are favorable for the electrocatalytic activity of the co-doped soft templated carbon felt. Based on current knowledge it seems that the enhancement of the surface has the most significant influence on the performance, but details of this observation have to be analyzed in more detail in the future.

## Conclusion

A new method was developed to synthesize carbon–carbon composite materials with an increased surface area and nitrogen and sulfur heteroatom content. These electrodes demonstrated promising activities for the positive half-cell reaction in the all-vanadium redox flow battery. Co-doping with nitrogen and sulfur can introduce a significant number of functional groups, which presumably increases the active sites for the VO^2+^/VO_2_^+^ redox couple and therefore substantially contributes to the increase of the battery performance. With the soft-templating approach a porous carbon–carbon composite felt with a significantly enhanced surface area (up to 20 times) was obtained. In case of the co-doped material the electrochemical evaluation exhibited a higher double-layer capacity indicating a higher surface area in contact with the electrolyte. This method does not rely on expensive precursors and thus enables an environmentally friendly way to achieve porous carbon electrode materials without the utilization of zinc chloride or other hazardous substances.

## Experimental

### Materials

2-Thiophenecarboxaldehyde (98%), pyrrole-2-carboxaldehyde (98%), phloroglucinol (≥99.0%), Pluronic^®^ F-127 and ethanol were purchased from MERCK. Hydrochloric acid (37%, Rotipuran^®^, p.a.) and ethanol (99.8%, p.a.) were purchased from Carl Roth^®^. All chemicals were used as obtained without any further purification. As a precursor material PAN-based carbon felts, which were received from Freudenberg Technology Innovation SE & Co. KG (Weinheim, Germany), were used.

Throughout this manuscript the untreated carbon felt is denoted as pristine. All further felts are abbreviated with the maximum temperature used during the carbonization step and the corresponding dopant.

### Material synthesis

To produce a suitable reference material, the pristine carbon felt was carbonized at 800 and 1000 °C under inert atmosphere (N_2_, 100%). The maximum temperature was held for 1 h before letting the furnace cool down.

To produce composite electrodes a soft-templating approach was utilized, following the synthesis route of Martinez de Yuso and co-workers [23]. Phloroglucinol (1,3,5-benzenetriol, C_6_H_6_O_3_; 0.219 g) as carbon source and Pluronic^®^ F-127 ((polyethylene oxide)-(polypropylene oxide)-(polyethylene oxide); 0.437 g) were dissolved in ethanol (40 mL), which was acidified before with HCl (0.3 mL, 37%). Then a solution of pyrrole-2-carboxaldehyde (0.166 g) in ethanol (5.5 mL) was added. In case of the co-doped carbon composite electrode 2-thiophenecarboxaldehyde (0.3 mL) as sulfur source was incorporated as well. The mixture was stirred for 1 min before the felts (size: 3.5 cm × 3.5 cm; average mass: 0.350 g) were soaked homogeneously with the combined solutions (20 mL per felt) in several petri dishes.

The solvent was let to evaporate at room temperature over 6–7 h, followed by thermopolymerisation in a furnace at 80 °C for at least 13 h, but not more than 15 h. Afterwards the carbon felt was thoroughly separated from the bulk material, which was scraped carefully off the petri dish. For the purpose of carbonization both materials were heated in incinerating dishes up to maximum temperatures of 800 and 1000 °C under a constant flow of nitrogen and with a heating rate of 2.5 °C/min. The tube furnace was allowed to cool down to room temperature after the maximum temperature was kept for 1 h. The composite electrodes were obtained with different mechanical stabilities. In comparison to the flexible pristine felt they appear rigid. With respect to their application in RFB batteries this could be a positive asset as this feature might generate a better contact between electrode and bipolar plate and reduce electrical resistances. All felts were covered with a visible black glossy layer.

### Structural characterizations

A scanning electron microscope (SmartSEM Supra 55VP, Carl Zeiss SMT Ltd.) with an acceleration voltage of 5–10 kV and an in-lens detector was utilized to obtain details of the surface structure. High-resolution images (Figure 2) were captured at several magnifications to investigate features at the microscale and the nanoscale. Energy-dispersive X-ray spectroscopy (EDX) analyses were performed with an X-Max 50 silicon drift detector (Oxford Instruments) at an acceleration voltage of 10 kV.

Nitrogen adsorption and desorption isotherms were recorded at 77 K using a high-resolution Micromeritics 3Flex instrument. Prior to the measurement the samples were degassed under vacuum at 120 °C for 4 h. The surface area was calculated according to the Brunauer–Emmett–Teller (BET) model.

Elemental analyses were accomplished using the VarioEL Organic Elemental Analyzer from Elementar Analysensysteme GmbH to determine the carbon, nitrogen, sulfur and hydrogen content. The average of the measured values was determined and listed in Table 2.

The elemental composition and the chemical state of the elements in the sample surfaces were determined by X-ray photoelectron spectroscopy (XPS) measurements (CLAM4 electron analyzer from Thermo VG scientific), using a Mg Kα source (1253.6 eV). For analysis, the peaks were fitted using Gaussian and Lorentzian functions with identical FWHM for each component of the same element after manual background subtraction [32].

### Electrochemical characterization

All electrochemical measurements were carried out with a Reference 600 potentiostat from Gamry Instruments operating in a three-electrode setup. A saturated calomel electrode (SCE) was used as reference electrode (243 ± 2 mV vs SHE) and a platinum electrode consisting of a 1 mm thick platinum piece (0.6 cm × 0.7 cm) represented the counter electrode. The respective carbon felts served as working electrodes and were pierced in their center with a 1 mm thick glassy carbon rod for contacting. For studying the VO^2+^/VO_2_^+^ redox reaction the electrolyte consisting of 100 mL of 0.2 mol/L vanadylsulfate (VOSO_4_, Sigma-Aldrich) dissolved in 2.0 mol/L sulfuric acid (H_2_SO_4_, for analysis, 96%) was utilized from Acros Organics. Prior to each measurement the electrolyte was purged with nitrogen for at least 15 min to make sure that there was no remaining dissolved oxygen in the solution. The homogeneous wetting of the felts was ensured by dipping them into the electrolyte solution for at least 30 min before measuring the samples. CV curves were measured with a scan rate of 2 mV/s and the scan limits were set to 0.3 and 1.2 V. The potentiostatic EIS experiments were implemented in a frequency range from 10^5^ to 10^−1^ Hz with an ac amplitude of 10 mV and a dc potential of 0.75 V vs SCE. The EIS data were fitted in the frequency range between 10^3^ and 10^0^ Hz. The value of the CPE was converted to *C*_DL_.
